# Follicle Structure Influences the Availability of Oxygen to the Oocyte in Antral Follicles

**DOI:** 10.1155/2011/287186

**Published:** 2011-11-17

**Authors:** A. R. Clark, Y. M. Stokes

**Affiliations:** ^1^Auckland Bioengineering Institute, The University of Auckland, Auckland 1142, New Zealand; ^2^School of Mathematical Sciences, The University of Adelaide, Adelaide, SA 5000, Australia

## Abstract

The ability of an oocyte to successfully mature is highly dependent on intrafollicular conditions, including the size and structure of the follicle. Here we present a mathematical model of oxygen transport in the antral follicle. We relate mean oxygen concentration in follicular fluid of bovine follicles to the concentration in the immediate vicinity of the cumulus-oocyte complex (COC). The model predicts that the oxygen levels within the antral follicle are dependent on the size and structure of the follicle and that the mean level of dissolved oxygen in follicular fluid does not necessarily correspond to that reaching the COC.

## 1. Introduction

A mammalian oocyte develops within a follicle in the ovary. The presence and growth of the follicle is likely to affect nutrient levels reaching the oocyte, and intrafollicular conditions may have an influence on the developmental competence of oocytes (the ability to mature, be fertilized, and develop into an embryo [1]) that does not become evident until some time after fertilisation [2]. The success of assisted reproduction technologies relies on developmentally competent oocytes being selected for treatment. The level of follicular vascularity, which directly affects the supply of nutrient to the follicle, has been linked to *in vitro* fertilisation (IVF) outcomes [3], and the nutrient composition of intrafollicular fluid has been studied as a prognostic parameter for selecting oocytes for IVF [4]. 

While traditional IVF uses oocytes matured *in vivo*, it can be desirable, or even necessary, to use immature oocytes which must first be matured in the laboratory, a process known as *in vitro* maturation (IVM). IVM is not widespread in humans, although it has potential to become a viable alternative to traditional IVF in cases where the mother is susceptible to ovarian hyperstimulation syndrome (OHSS) or polycystic ovary syndrome (PCOS) [5–8]. IVM is however used routinely in ruminants such as cattle where management of reproduction is an important industry concern [1, 9]. The development of IVM systems that optimise the successful development of the embryo has long been identified as essential in both commercial and research settings [10]. However, the success of IVM suffers from differences between the nutrient composition in culture media compared with *in vivo* conditions [11, 12]; hence, an understanding of intrafollicular conditions is critical for successful oocyte selection and maturation regardless of species.

**Figure 1:**
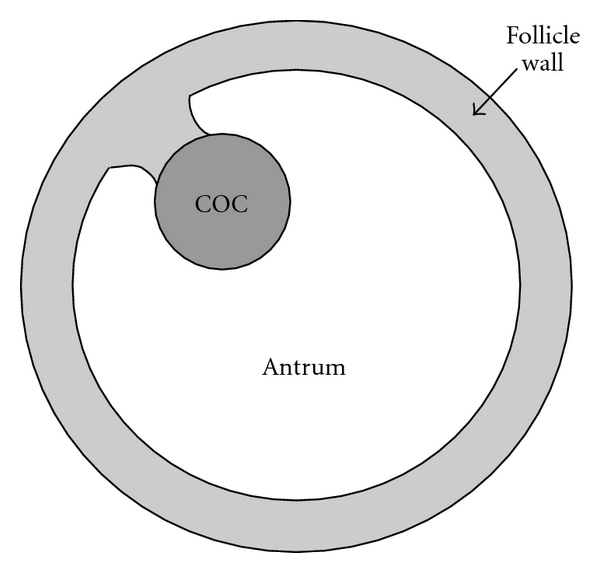
The antral follicle with the follicle wall, the antrum, and the COC labelled. In some follicles (particularly smaller follicles), the COC sits partially within the follicle wall; however, in many follicles it rests within the antrum, either next to the follicle wall or attached to it by a “stem” of granulosa cells as shown here.

The avascular part of the follicle structure is shown schematically in the antral stage of development in Figure 1. This structure consists of the follicle wall (made up of mural granulosa cells), surrounding a region comprised of follicular fluid (the antrum). The oocyte and its surrounding layers of cumulus cells, known collectively as the cumulus-oocyte complex (COC), are eccentrically placed within the follicle. In some follicles (particularly smaller follicles), the COC sits partially within the follicle wall; however, in many follicles it rests within the antrum, either next to the follicle wall or attached to it by a “stem” of granulosa cells. Surrounding the avascular part of the follicle is the basal lamina and the theca externa and theca interna (not shown in Figure 1). The vascular supply to the follicle, and so the supply of nutrients to the oocyte, lies in theca layers around the perimeter of the follicle.

The composition of follicular fluid varies between follicles and depends on their size and structure [4, 13]. Each antral follicle has a unique follicular fluid composition, and, despite several experimental studies, it is still not clear what constitutes a follicular environment that allows oocytes to develop successfully [2, 4]. Without a quantitive description of *in vivo* conditions the development of *in vitro* systems must rely to some extent on a “trial and error” approach. Mathematical modelling of nutrient transport in the follicle provides understanding of how the follicular environment affects the levels of nutrient seen by the oocyte. Previous mathematical models of nutrient transport in the antral follicle focus on investigation of oxygen (O_2_) transport [14, 15]. These studies treat the follicle wall as a homogeneous mass of cells and do not explicitly consider the COC, which is assumed to lie within, and behave in the same manner as, the follicle wall. We explicitly include the COC in our model under the hypothesis that the oocyte protrusion into the follicle may affect its nutrient environment. To our knowledge, this explicit inclusion of the COC within the follicle has not been considered in modelling studies.

Several experimental studies indicate that perifollicular vascularity is associated with oocyte developmental competence [2, 16]. This has in turn been associated with the dissolved O_2_ content of follicular fluid [16], although this association has been disputed [17]. While O_2_ is clearly essential for successful oocyte maturation, very high O_2_ levels can result in an increased presence of, for example, reactive oxygen species (ROS), which can be damaging to the oocyte [18]. Studies examining the effect of O_2_ concentration during *in vitro* maturation of oocytes across several species have not yielded a consensus on the optimal O_2_ concentration for oocyte maturation, due to conflicting results [19–24]. Here we present a mathematical model which relates perifollicular O_2_ concentration to follicular fluid O_2_ concentration and the O_2_ concentration reaching the COC in antral follicles. 

Thus, this model is the first of its kind to explicitly include a COC, which allows prediction of the nutrient environment near the oocyte itself, as well as whole follicle measures. The model predicts that the O_2_ levels within the antral follicle are dependent on the size and structure of the follicle and that the average level of dissolved O_2_ in follicular fluid does not necessarily correspond to that reaching the COC. 

## 2. Mathematical Model

### 2.1. Model Geometry

**Figure 2:**
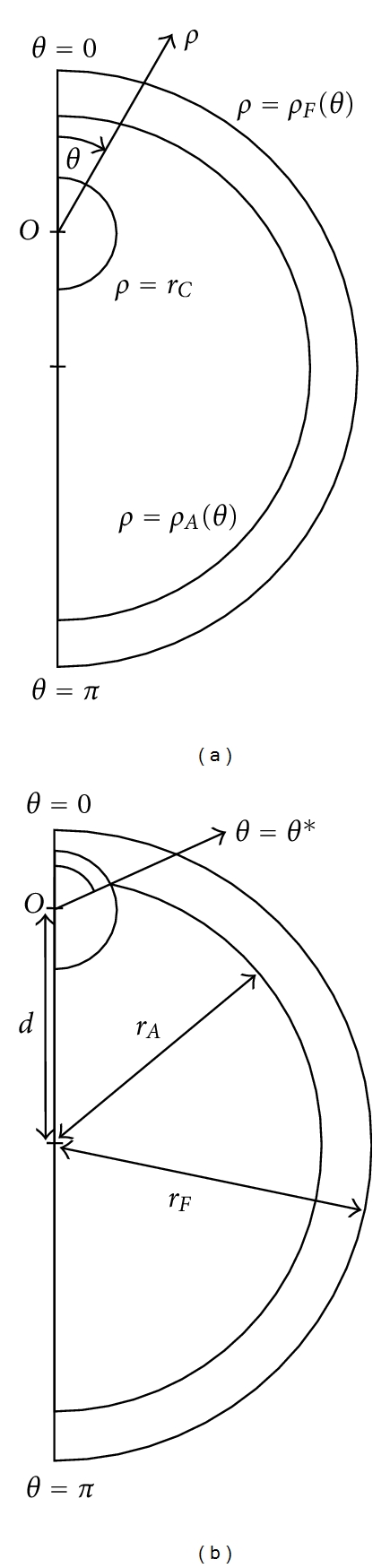
The model geometry used to represent the antral follicle. This geometry is described using polar coordinates (*ρ*, *θ*) with axial symmetry. The origin of the geometry is taken to be the centre of the COC, and thus the origin varies with respect to the antrum and the follicle wall. The geometry is shown for (a) the COC entirely within the antrum and (b) the COC partially enclosed within the antrum and partially within the follicle wall, intersecting the inner boundary of this layer at *θ* = θ*.

The model proposed here considers the follicle wall, the antrum, and the COC as three separate regions. The antral follicle is approximated as an axially symmetric structure, with rotational symmetry about the line that passes through both the centre of the follicle and the centre of the COC. The model geometry is shown in Figure 2 with the COC in two different positions within the follicle. The origin is chosen as the centre of the COC (labelled *O* in Figure 2) and is distance *d* from the centre of the follicle. An appropriate coordinate system is (*ρ*, *θ*), where *ρ* ≥ 0 is radial distance from the origin and *θ* is the angle shown in Figure 2(a). The axially symmetric geometry is described by considering 0 ≤ *θ* ≤ *π*. As the origin is at the centre of the COC, the boundary at the edge of the COC is described by *ρ* = *r*_*C*_, where *r*_*C*_ is the constant radius of the COC. The boundaries of the antrum, *ρ*_*A*_(*θ*), and the follicle, *ρ*_*F*_(*θ*), are

(1) $$\;\begin{array}{cc} \rho_{A}\left(\theta \right) & =\sqrt{r_{A}^{2}-d^{2}\sin\left(\theta \right)}-d\cos\left(\theta \right),\;\theta^{*}\leq \theta \leq \pi ,\\ \rho_{F}\left(\theta \right) & =\sqrt{r_{F}^{2}-d^{2}\sin\left(\theta \right)}-d\cos\left(\theta \right),\;0\leq \theta \leq \pi , \end{array}\;$$

where *r*_*A*_ is the radius of the antrum and *r*_*F*_ the radius of the follicle as measured from the center of the follicle. When the COC is partially embedded in the follicle wall, θ* is the value of *θ* at which the COC and the antrum intersect and

(2) $$\theta^{*}=\text{arc}\;\cos\left(\frac{r_{A}^{2}-r_{C}^{2}-d^{2}}{2dr_{C}}\right).$$

If the COC is completely within the antrum, then θ* = 0 in (1).

The rate of follicle growth is assumed to be slow compared with the rate of O_2_ transport within the follicle. Thus, a steady-state model at each stage of follicle development is appropriate. The antral fluid is formed from transudate from blood plasma and also secretions from granulosa cells, cumulus cells, and the oocyte [25]; this may result in fluid motion and hence mixing. It is assumed that any fluid motion is negligible and so the dominant mechanism of O_2_ transport throughout the follicle is by diffusion. In the antrum itself, it is assumed that O_2_ consumption is negligible; therefore, O_2_ concentration in the antrum, *c*_*A*_, satisfies Laplace's equation

(3) $$\frac{\partial }{\partial \rho }\left(\rho^{2}\frac{\partial c_{A}}{\partial \rho }\right)+\frac{1}{\sin\theta }\frac{\partial }{\partial \theta }\left(\sin\theta \frac{\partial c_{A}}{\partial \theta }\right)=0.$$

In the follicle wall we assume that the cumulus cells can be treated as a homogenous layer of intra- and extracellular matter, as in previous models of O_2_ transport in the antral follicle [14, 15]. Therefore, the O_2_ concentration in the follicle wall, *c*_*G*_, satisfies

(4) $$\frac{D_{G}}{\rho^{2}}\frac{\partial }{\partial \rho }\left(\rho^{2}\frac{\partial c_{G}}{\partial \rho }\right)+\frac{D_{G}}{\rho^{2}\sin\theta }\frac{\partial }{\partial \theta }\left(\sin\theta \frac{\partial c_{G}}{\partial \theta }\right)=\left(1-\alpha_{G}\right)q_{G},$$

where *D*_*G*_ is the diffusion coefficient of O_2_ in the follicle wall, *α*_*G*_ is the volume fraction of extracellular material in the follicle wall (the ratio of extracellular to total volume), and *q*_*G*_ is the rate of O_2_ uptake by granulosa cells (amount per unit volume of granulosa cell mass per unit time). Experimental determination of oxygen consumption by ovine granulosa cells and murine embryos has indicated little dependence on O_2_ concentration, except at very low concentrations [26, 27]. The function *q*_*G*_ is therefore assumed to be independent of O_2_ concentration as in previous studies of O_2_ transport in granulosa cells, embryos, and follicles [14, 15, 26, 27].

Consumption of O_2_ by the COC is incorporated into this model via a flux condition at the COC boundary

(5) $$\begin{array}{c} D_{G}\frac{\partial c_{G}}{\partial \rho }\left(r_{C},\theta \right)=q_{\text{COC}},\;0\leq \theta \leq \theta^{*},\\ D_{A}\frac{\partial c_{A}}{\partial \rho }\left(r_{C},\theta \right)=q_{\text{COC}},\;\;\theta^{*}\leq \theta \leq \pi , \end{array}$$

where *D*_*A*_ is the diffusion coefficient of O_2_ in the antrum and *q*_COC_ is the rate of O_2_ uptake by the COC (amount per unit COC surface area per unit time). As with the rate of O_2_ uptake by granulosa cells, the rate of O_2_ uptake by the COC is assumed constant.

 At the boundary between the follicle wall and the antrum continuity of flux and concentration hold. Symmetry conditions apply at *θ* = 0 and *θ* = *π* in both the follicle wall and the antrum. 

The flux of O_2_ into the follicle at the boundary *ρ*_*F*_(*θ*), the edge of the follicle, is represented by

(6) $$D_{G}\frac{\partial c_{G}}{\partial n_{F}}\left(\rho_{F}\left(\theta \right),\theta \right)=h_{p}\left(c_{P}-c_{G}\left(\rho_{F}\left(\theta \right),\theta \right)\right),$$

where *c*_*P*_ is the O_2_ concentration in the blood vessels surrounding the follicle and ∂*c*_*G*_/∂*n*_*F*_ is the concentration gradient in the direction of the outward unit normal vector to the follicle surface. The parameter *h*_*p*_ is the transfer coefficient for this problem and quantifies the ease of O_2_ transport from the vascular network of the ovary into the follicle.

Model parameters for the bovine antral follicle and a parameter sensitivity analysis are given in Table 1. The diffusion coefficients of O_2_ in the antrum and the follicle wall are assumed to be that of O_2_ in physiological saline [28]. An average bovine preovulatory follicle diameter is 15–18 mm [29]. However, bovine ovulatory follicle radius is highly variable, depending on the number of follicles ovulated, breed, age, and any hormones administered. A study using ultrasonography classified follicles by animal age, the number of follicles ovulated, and the year (2004–2006) and showed bovine preovulatory follicle diameters ranging from 8 to 30 mm (radii of 4–15 mm), with a mean in each group considered ranging from 13.9 to 17.1 mm (radius 6.95–8.55 mm) [29]. Therefore, a range of follicle radius values, *r*_*F*_, up to 15 mm were considered to allow consideration of antral follicles through development to ovulation. There is no apparent relationship between the radius of a follicle and the thickness of its follicle wall in bovine antral follicles [30, 31], and there appears to be considerable variation in granulosa cell numbers between follicles [15]. However, a relationship between granulosa cell number (*n*_*G*_) and follicle radius has been determined in humans by Redding et al. [15] as

(7) $$n_{G}=\frac{a}{1+be^{2cr_{F}}},$$

where *a*, *b*, and *c* are constants. The antrum radius, *r*_*A*_, is then calculated using

(8) $$\text{Follicle wall volume}=\frac{n_{G}v_{G}}{1-\alpha_{G}}=\frac{4\pi r_{F}^{3}}{3}-\frac{4\pi r_{A}^{3}}{3},$$

where *v*_*G*_ is the volume of a granulosa cell. The volume fraction of extracellular material in the follicle wall of a bovine follicle, *α*_*G*_, is not well determined. A value of *α*_*G*_ = 0.3 is thought to be typical of volume fractions in the follicle wall of mammalian follicles [14, 15] and is used in previous studies of O_2_ transport in the antral follicle. However, as discussed in those studies, accurate experimental determination of this volume fraction would benefit models of this type. The magnitude of the parameter *h*_*p*_ is unknown and so a range of values for this parameter was considered; model solutions were insensitive to *h*_*p*_ for *h*_*P*_ > 0.01 m/s. 

In the special case that the COC lies at the centre of the follicle the model described by (3)–(6) is analytically solvable. Although this is an unlikely physical scenario, especially in large follicles, it provides validation of numerical solution procedures. Also, as will be discussed in Section 3 of this study, it provides a “worst case” solution where predicted O_2_ concentrations reaching the COC are lowest. The model of Redding et al. [14, 15], which incorporates the COC mass into the follicle wall is also considered here, holding total cell mass (COC plus granulosa cells) constant between models. This model is also analytically solvable and provides a “best case” scenario where predicted O_2_ concentrations reaching the COC are highest. When numerical solutions were necessary, they were obtained using the finite element solver COMSOL Multiphysics (Version 3.3, COMSOL AB, 2006). The solutions are shown here in two cases: (1) with the COC resting on the inner surface of the follicle wall, and (2) with the COC within the antrum one COC radius (*r*_*C*_) away from the follicle wall.

## 3. Results

**Figure 3:**
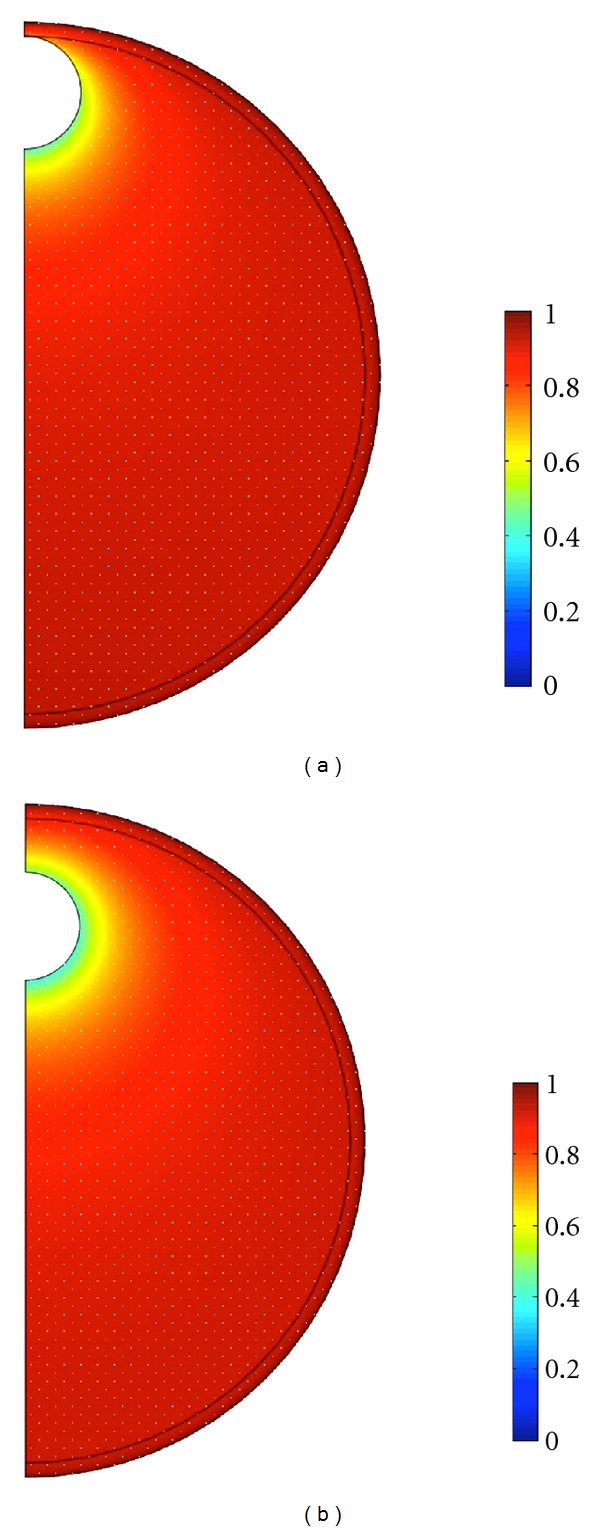
The O_2_ concentrations (normalized by *c*_*P*_) in a follicle of 1 mm radius when (a) the COC rests on the inner boundary of the follicle wall and (b) the COC is a distance of one radius (*r*_*C*_) away from the follicle wall. Explicit inclusion of the COC in the model shows a decrease in O_2_ concentration within the antrum in the vicinity of the COC.

When the COC cell mass is incorporated into the follicle wall, the predicted O_2_ concentration in the antrum is constant and equal to that at the inner boundary of the follicle wall, which is little less than the perifollicular concentration *c*_*P*_. However, when the COC is explicitly included in modeling, there is a significant decrease in O_2_ concentration in the vicinity of it. Figure 3 shows O_2_ concentrations (normalized by *c*_*P*_) in a follicle of 1 mm radius for the COC in two different positions in the antrum. 

**Figure 4:**
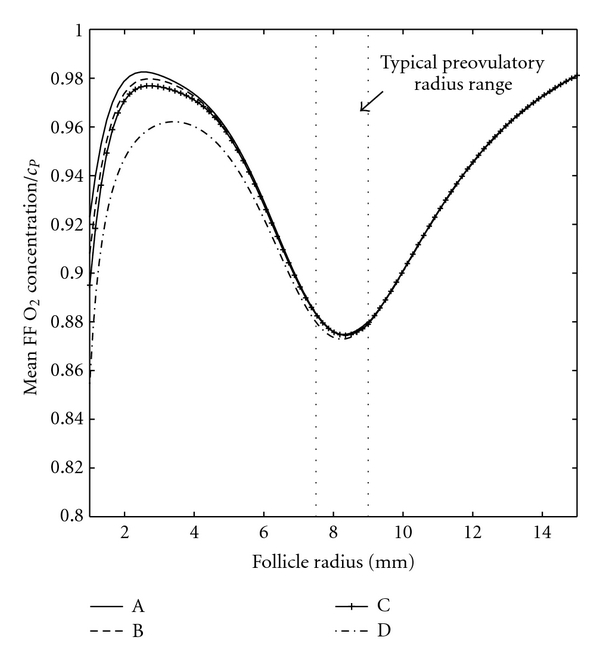
Mean follicular fluid (FF) O_2_ concentration versus follicle radius for (A) the COC incorporated into the follicle wall, (B) the COC resting on the inner boundary of the follicle wall, (C) the COC a distance of one radius (*r*_*C*_) away from the follicle wall, and (D) the COC at the centre of the follicle wall.

It is straightforward for experimentalists to measure the mean dissolved O_2_ concentration in follicular fluid, whereas it is very difficult to measure the O_2_ concentration in the vicinity of the COC, particularly *in vivo*. This mean O_2_ concentration, as predicted by our model, is plotted versus follicle radius in Figure 4 for (A) the COC incorporated into the follicle wall, (B) the COC resting on the inner boundary of the follicle wall, (C) the COC a distance of one radius (*r_C_*) away from the follicle wall, and (D) the COC at the centre of the follicle. Case (A) is the model considered by Redding et al. [14, 15] for human follicles, and our results correspond to that model when the human parameter values from those studies are used. Cases (A) and (D), respectively, provide a maximum and a minimum for predictions of mean follicular fluid O_2_ concentration and solutions do not differ substantially in this case. A typical ovulatory follicle radius range [29] is shown, and this corresponds to a local minimum in O_2_ concentration. 

**Figure 5:**
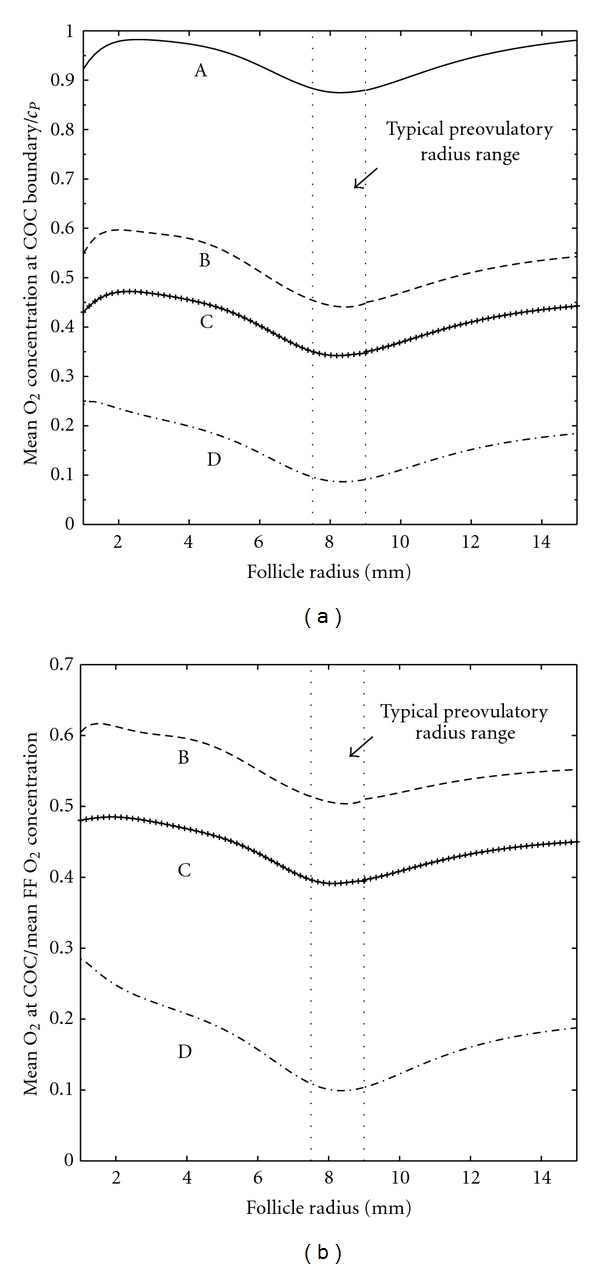
(a) Mean O_2_ concentration at the surface of the COC versus follicle radius for (A) the COC incorporated into the follicle wall, (B) the COC resting on the inner boundary of the follicle wall, (C) the COC a distance of one radius (*r_C_*) away from the follicle wall, and (D) the COC at the centre of the follicle wall. (b) The ratio of mean O_2_ concentration at the surface of the COC to mean follicular fluid (FF) O_2_ concentration plotted against follicle radius for the cases when the COC is explicitly included in modelling (cases B–D).

However, model predictions of the mean O_2_ concentration at the COC surface in each case differ substantially from predictions of mean O_2_ concentration in follicular fluid (Figure 5(a)). It is clear that the closer the COC to the centre of the antrum, the lower the O_2_ concentration at its surface.  Figure 5(b) shows the ratio of the mean O_2_ concentration in follicular fluid to the mean O_2_ concentration at the COC surface in the cases where the COC is explicitly included in modelling (cases B, C, D). When the COC rests on the follicle wall (case B), the level of O_2_ reaching the COC is just 60% of the follicular fluid average, and this ratio decreases as the COC gets closer to the centre of the antrum. 

The model is highly sensitive to the diffusion coefficients *D*_*A*_ and *D*_*G*_. There is some discussion in the literature about the appropriate diffusion coefficient for oxygen in tissues (see [32] for a thorough discussion of the literature regarding this matter). If it were the case that either diffusion coefficient was lower than that in physiological saline, this would reduce O_2_ concentrations throughout the antrum and at the COC surface but would not change the qualitative nature of results. Similarly changes to *q*_COC_ and *r*_*C*_, properties of the COC to which the model is sensitive, do not change the qualitative nature of results. 

## 4. Discussion 

Here we have described the construction of a mathematical model to describe O_2_ transport in the antral follicle which explicitly includes a COC which may protrude into, or lie entirely within, the antrum. This model enables linking of follicular O_2_ concentration, a measurable quantity which has been proposed as a prognostic parameter in assisted reproduction technologies, and the O_2_ environment of the oocyte. Model results show that the structure of the follicle is important in determining O_2_ availability to the oocyte, and so this structure is also likely to affect developmental competence, a factor which may be important when selecting oocytes for assisted reproduction. 

Model predictions of mean O_2_ concentration in follicular fluid show the same form as those obtained using the model of O_2_ transport in the antral follicle described by Redding et al. [14, 15], which did not explicitly include a COC. Results from these studies, and the work described in this paper, give understanding of the level of dissolved O_2_ in an antral follicle at different stages of development. At the start of the antral stage predicted mean O_2_ concentration rises rapidly with follicle radius to a local maximum at a radius of 2-3 mm (Figure 4). At this stage follicle growth is predominantly due to antrum formation, rather than granulosa cell proliferation. An increasing follicular surface area and decreasing follicle wall thickness (as a fraction of follicle radius) allow more O_2_ to enter the follicle resulting in the observed increase in O_2_ concentration with follicle radius. Later in follicle growth, granulosa cell proliferation increases the thickness of the follicle wall resulting in a decrease in O_2_ concentration within the antrum. 

There is a local minimum in O_2_ concentration predicted by the model at around 8 mm radius (16 mm diameter) which falls within the range of typical diameters for bovine ovulatory follicles. Beyond that radius the O_2_ concentration within the follicle steadily rises toward a global maximum which, in the case of mean follicular fluid O_2_ concentration, is close to plasma O_2_ concentration. This implies that larger than average preovulatory follicles, provided that they are sufficiently vascularised, will see a larger than average O_2_ concentration. Whether this is beneficial or detrimental to their subsequent development when fertilised is likely to depend on the associated level of ROS, which is known to increase at high O_2_ concentrations. In bovine IVM it has been shown that the developmental potential of an oocyte increases with follicle (and oocyte) diameter in follicles up to 15 mm in diameter [33]. In studies using hormonal treatment to induce ovulation before artificial insemination, the pregnancy rate increased with follicle diameter to, approximately, 12–15 mm in diameter and then decreased as follicle size increased further [34, 35]. However, changes in pregnancy rate with follicle diameter were not detected in spontaneously ovulating cows [34]. 

An important difference between our model and previous models is the explicit inclusion of the COC. This allows the position of the COC within the follicle to be altered to determine the effects of follicle geometry on the O_2_ environment within the follicle. Average O_2_ concentrations in follicular fluid are higher the closer the COC sits to the follicle wall, and neglecting the COC from models will necessarily overestimate the O_2_ seen by the COC, unless the COC is completely incorporated into the follicle wall. Our model suggests that the position of the COC within the follicle plays a significant role in determining the O_2_ concentration seen by the oocyte and that follicle structure should be taken into account, along with average dissolved follicular fluid O_2_ concentration, in order to understand the effect of O_2_ concentration on the developmental competence of the oocyte. The influence of follicle geometry on the O_2_ availability to the COC may give some indication as to why the dissolved O_2_ content of follicular fluid has not been conclusively linked to developmental competence of oocytes [16, 17]. 

In terms of cattle breeding or even human IVM systems that aim to replicate the *in-vivo* environment, the finding that average follicular fluid oxygen concentration is likely to be considerably higher than that reaching the COC should be taken into account when considering optimal incubator settings. The geometry of an *in vitro* environment may also influence the availability of O_2_ to the COC—for example, the volume of fluid in which the COC is matured may alter O_2_ availability—and this may need to be accounted for when determining incubator settings for IVM. 

Figure 3 shows that the drop in oxygen concentration across the mural granulosa follicle wall is small while there is a significant decrease in follicular oxygen concentration in the vicinity of the COC. This implies that the granulosa cells do not play a large role in determining follicular fluid oxygen concentrations so sparing oxygen for use by the maturing oocyte. The parameter describing oxygen uptake by mural granulosa is a factor of ten lower than that describing oxygen consumption by cumulus cells (the cells directly surrounding the oocyte) given by Clark et al. [32]. There is *in vitro* evidence from murine [36, 37] and bovine [38] studies that, in the absence of follicle stimulating hormone (FSH), the metabolism of cumulus and granulosa cells differs significantly. This suggests differing roles for the granulosa and cumulus cells in supporting the developing oocyte. Models show that both the cumulus cells [32] and the granulosa cells (modelled here) appear to spare oxygen, which is necessary for ATP production within the oocyte. However, cumulus cells must provide glucose metabolites such as pyruvate to the oocyte to support its metabolism (as the immature oocyte has limited capability for metabolising glucose [39]), so that a fine balance of sparing oxygen for the oocyte and utilising oxygen to produce pyruvate must be met in these cells. On the other hand the metabolism of the mural granulosa is able to be largely anaerobic which allows as much oxygen as possible through to the developing follicle. It is known that removal of the cumulus cell matrix from the oocyte is detrimental to oocyte development [40, 41] and the metabolic support that cumulus cells provide to the oocyte makes inclusion of the whole COC in IVM systems desirable [5]. On the other hand, it seems that mural granulosa cells are important for folliculogenesis rather than providing the oocyte with nutrients [36]. Mathematical models of this type applied to other important nutrients, for example, may provide further insight into the differing roles of these two cell types in oocyte development that may lead to improvements in *in vitro* handling of oocytes and COCs. 

Neither this model, nor previous models of O_2_ transport in the antral follicle [14, 15, 26], account explicitly for angiogenesis. Angiogenesis occurs around the follicle in response to the release of stimulating chemicals released from follicular cells [42, 43]. One of these chemicals, vascular endothelial growth factor (VEGF), is known to have increased expression as a response to hypoxia [43]. In addition, at least in pigs, angiogenesis appears to begin in medium-sized follicles [43]. This coincides with a stage of development when models of nutrient transport in the follicle suggest a significant drop in both dissolved follicular fluid O_2_ concentrations and O_2_ availability to the oocyte. This lends support to the suggestion that follicular growth is limited by both the nutrient environment within the follicle [14] as well as the development of an adequate vascular supply [43]. An unevenly distributed vascular supply to the follicle is not expected to have much effect on the uniformity of O_2_ concentration within the follicular fluid, unless there are large “gaps” in the vascular supply. This is due to the small size of O_2_ molecules meaning that they diffuse readily across membranes and through extracellular material. However, the distribution of vascularisation may have implications for the transport of larger molecules in the follicle. 

The data for cell number versus follicle radius used in this study is for human antral follicles, whilst other model parameters are determined for bovine follicles. The location of the local maximum for O_2_ concentrations in follicular fluid and at the COC could be predicted more accurately with data on bovine granulosa cell number versus follicle radius should this become available. Similarly, with accurate parameterization, this model can be applied to humans or other mammalian species, as well as for other substrates that diffuse easily across cell membranes. 

The model represents an idealised spherical follicle containing a spherical COC and does not account for O_2_ consuming cells in any “stem” attaching the COC to the follicle wall, nor the structural and functional changes in the COC prior to ovulation. A “stem” of cumulus cells will slightly reduce the O_2_ concentration reaching the COC. Therefore, this idealised geometry will slightly overestimate the O_2_ environment seen by the COC. In the final stages of maturation, immediately before ovulation, the cumulus cell mass expands, so the space between cumulus cells increases and the extracellular material surrounding these cells becomes mucinous [40]. Computation of oxygen concentrations at this final stage of maturation may require appropriate changes to the model geometry and, probably, the rate of oxygen diffusion through the cumulus matrix toward the oocyte, that is, the diffusion coefficient in this region. Finally the model assumes that COC oxygen consumption is constant throughout maturation. An increase in oxygen demand by the COC, for example, late in development, would lower follicular oxygen levels significantly (see Table 1) which may restrict oxygen availability to the oocyte.

## 5. Summary 

We have described a mathematical model of O_2_ transport in the antral follicle so as to link the O_2_ concentration in follicular fluid (measured using fluid aspirated from the ovary) with the O_2_ environment of the COC itself, taking into account the structure of the follicle. This model differs from previous models of nutrient transport in the ovarian follicle [14, 15, 26] in its explicit inclusion of the COC. It predicts the O_2_ concentration seen by the oocyte to differ significantly from the mean concentration in the follicle antrum showing that follicle structure as well as perifollicular O_2_ supply can affect the ability of an oocyte to successfully mature. Our model enables a better informed use of follicular fluid O_2_ concentration as an indicator of developmental competence of oocytes. Hence, it enables better informed selection of oocytes for use in assisted reproduction technology programs and better choice of culture conditions for *in vitro* maturation of oocytes. 

## Figures and Tables

**Table 1:** Nominal model parameters and sensitivity analysis. The sensitivity analysis is carried out by considering the nominal parameter values as the baseline (with the COC centrally located within the follicle) and calculating the change in the mean O_2_ concentration at the COC boundary (*r*_*C*_) for a 10% increase or decrease in each parameter from the baseline.

Parameter	Value	Reference	+10%	−10%
**α*_G_*	0.3 (no units)	[14, 15]	−1.5%	+1.4%
*C_P_*	0.128 (mol m^−3^)	[14]	+41%	−41%
*D_A_*	2.5 × 10^−9^ (m^2^ s^−1^)	[28]	+25%	−30%
*D_G_*	2.5 × 10^−9^ (m^2^ s^−1^)	[28]	+3.1%	−3.7%
*h_P_*	0.1 (m s^−1^)	Variable (see text)	See discussion	See discussion
*q_G_*	0.0363 (mol m^−3 ^s^−1^)	[14]	−3.1%	+3.1%
*q*_COC_	1.62 × 10^−6^ (mol m^−2 ^s^−1^)	From [44], units converted using *r*_*C*_	−28%	+28%
*r_F_*	1 × 10^−3^ (m)	See text	+1.9%	−4.5%
*r_C_*	160 × 10^−6^ (m)	[45]	+30%	−37%
*v_G_*	1.14 × 10^−15^ (m^3^)	[15]	−6.9%	+6.2%
*a*	43.1 × 10^6^	[15]	−6.9%	+6.2%
*b*	281.6	[15]	+5.6%	−7.6%
*c*	−387.3	[15]	−5.4%	+4.6%
